# A reliability generalization meta-analysis of self-report measures of statistics anxiety

**DOI:** 10.3389/fpsyg.2025.1675957

**Published:** 2026-01-23

**Authors:** Emine Ören Özdemir, Ibrahim Yildirim

**Affiliations:** 1 Department of Educational Sciences, Gaziantep University, Gaziantep, Türkiye; 2 Ministry of National Education, Gaziantep, Türkiye

**Keywords:** reliability generalization, meta-analysis, reliability induction, statistics anxiety, reliability

## Abstract

**Objective and Method:**

In this study, it was aimed to obtain a general reliability coefficient for each of the statistics anxiety scales. For this purpose, Web of Science, ERIC and Scopus databases and Google Scholar search engine were searched according to certain criteria and 84 Cronbach’s alpha coefficients reported for the whole scale were reached. Reliability generalization meta-analysis method was applied to the obtained alpha coefficients within the scope of meta-analysis, which is a quantitative method.

**Results and Conclusion:**

The mean value of the alpha coefficients for which the transformation formula was applied was found to be .927 under the random effects model, and the findings were statistically significant. In addition, the mean alpha value of each statistics anxiety scale was .931 for STARS, .917 for SAS, .918 for SAS-10 and .951 for WAESTA. Analog to the ANOVA and meta-regression analyses were conducted to reveal the heterogeneity of alpha coefficients in the overall analysis. Analog to ANOVA was applied for five different categorical variables, and according to the findings, it was observed that the mean alpha value differed statistically significantly depending on the scale type variable. Moreover, it was found that the mean scale score and the standard deviation of the mean scale score were statistically significant predictors of the mean alpha value.

## 1 Introduction

Statistics is a branch of science that covers the processes of making inferences from numerical data related to the organization, analysis or interpretation of data collected in daily life or school life. Statistics, which is widely used, has become a branch of science utilized in many different fields such as engineering, health sciences, social sciences and natural sciences (Akdeniz, 2015). An individual who will conduct a scientific study is expected to have some competencies in order to collect data, to bring these data together in a certain order and to interpret them correctly (Erkuş, 2011). In order to have these competencies, statistics education has become a necessity for individuals receiving undergraduate and graduate education (Onwuegbuzie, 1993).

The fact that statistics course is taught as a compulsory course to undergraduate and graduate students has brought along the need to examine the factors of success or failure in this course. Success in statistics course is affected by affective factors as well as cognitive factors (Yaşar, 2014). Feedback about the course (Williams, 2012), difficulties encountered in the course (Feinberg and Halperin, 1978), and past experiences associated with the mathematics course (Maysick, 1985) are among the cognitive factors affecting success in statistics course. The factors affecting the success of this course, such as the past academic experiences of each student taking the course, negative attitudes towards the teacher teaching the course, and the perception of interpreting numerical data paved the way for the formation of concerns about the course (Baloğlu, 2017). As a result of the formation of such concerns about the statistics course in individuals, the concept of “statistics anxiety” was added to the literature. Onwuegbuzie (1993) defines statistics anxiety as the state of anxiety that occurs when an individual encounters statistical processes such as organizing, analyzing or interpreting data at any time.

Since the ability to interpret numerical data in statistics is similar to the content of the mathematics course, the first studies on statistics anxiety tried to explain it with mathematics anxiety (Schacht and Stewart, 1990). There are also studies in which the relationship between statistical course success and situational anxiety was tried to be explained (Bendig and Hughes, 1954; Fisch, 1971). In this case, in the most general sense, statistical anxiety is examined under three subcomponents: situational causes, environmental causes and characterological causes (Onwuegbuzie, 1993). Situational causes of statistics anxiety include the role of the instructor teaching the statistics course (O’Bryant et al., 2021; Tonsing, 2018), pedagogical behaviors (Asare, 2023), course feedback (Williams, 2012), course content and pace of the course (Pan and Tang, 2004). Environmental causes of statistics anxiety are classified as the age (Bui and Alfaro, 2011), gender (Edirisooriya and Lipscomb, 2021), and past experiences with mathematics (Maysick, 1985). Under the characterological causes of statistical anxiety, individual characteristics such as academic motivation, learning styles (Kesici et al., 2011), self-perception (Esnard et al., 2021), self-identity (Najmi et al., 2018), and academic resilience (Ali and Gaber, 2022) stand out.

### 1.1 Statistics anxiety scales

Statistics anxiety scales have been developed by scientists in order to determine the level of this anxiety and to take measures for the result. Among these scales, STARS developed by Cruise et al. (1985) and SAS developed by Vigil-Colet et al. (2008) are the most widely used statistics anxiety scales worldwide. In addition to these scales, the SAS-10 scale, which was revised by Pretorius and Norman (1992) from the Mathematics Anxiety Scale developed by Betz (1978) to a statistics anxiety scale, and the WAESTA scales developed by Faber et al. (2018) are also widely used statistics anxiety scales. These scales have been adapted to many cultures and are widely used. There are also the SAM scale developed by Earp (2007) and the SAI scale developed by Zeidner (1991), which are not widely used.

### 1.2 Statistics anxiety rating scale (STARS)

STARS, developed by Cruise et al. (1985) on a five-point Likert scale, is the most widely used statistics anxiety scale. As a result of the factor analysis conducted, the STARS scale consisting of 51 items and 6 subscales was obtained. Worth of Statistics subscale consists of 16 items, Interpretation Anxiety subscale consists of 11 items, and Test and Class Anxiety subscale consists of 8 items. In addition, Computation Self-Concept subscale consists of 7 items, Fear of Asking for Help subscale consists of 4 items, and finally Fear of Statistics Teacher subscale consists of 5 items. When examining the subscales of STARS, it is seen that the first three subscales (WS, IA, TCA) are related to statistical anxiety, while the other three subscales (CSC, FAH, FST) are related to statistical competence. The internal consistency coefficient for the reliability of STARS for the sample of 537 participants was reported as 0.96 and the test–retest reliability coefficient was reported as 0.76, and the internal consistency coefficients of the subscales were 0.94, 0.87, 0.68, 0.88, 0.89 and 0.80, respectively.

### 1.3 Statistical anxiety scale (SAS)

In the SAS developed by Vigil-Colet et al. (2008), it was thought that the subscales of STARS other than statistics anxiety (fear of statistics teacher, self-perception, value of statistics) were not suitable for the purpose of measuring statistics anxiety. It was thought to be more useful since it has fewer items than STARS. Exploratory factor analysis was applied to ensure the validity of the SAS, which is a five-point Likert-type scale consisting of 24 items, and as a result of the analysis, it was decided that the scale had three subscales. Each of these subscales consists of 8 items. The factor loadings of the items in the first subscale vary between 0.88 and 0.57, the factor loadings of the items in the second subscale vary between 0.92 and 0.50, and the factor loadings of the items in the third subscale vary between 0.89 and 0.34. These subscales were named as Examination Anxiety, Asking for Help Anxiety and Interpretation Anxiety. The alpha coefficient for the sample of 159 undergraduate students for whom the SAS was developed was reported as 0.911. In addition, the alpha coefficients of the subscales of the SAS were determined as 0.87, 0.92 and 0.82, respectively.

### 1.4 WAESTA scale

The WAESTA scale developed by Faber et al. (2018) consists of 17 items. The aim was to create a measurement tool that is simpler and free from conceptual limitations than the scales previously developed in this field. In order to gather validity evidence based on internal structure of the scale, the factor loadings of each item were calculated using principal component analysis and it was seen that the scale consisted of three subscales. These subscales consist of the Worry subscale, Avoidance subscale, and Emotional subscale. Among these subscales, the factor loadings of the items belonging to the Worry subscale (consisting of eight items) ranged between 0.49 and 0.74, and the factor loadings of the items belonging to the Avoidance subscale (consisting of four items) ranged between 0.49 and 0.76. Finally, the factor loadings of the items belonging to the subscale named Emotional cognition (consisting of five items) vary between 0.53 and 0.70. Of the three subscales, The internal consistency coefficient for the sample in which the measurement tool was developed was reported as 0.94 and the split-half reliability coefficient was reported as 0.91.

### 1.5 The statistics anxiety scale (SAS-10)

The SAS-10 was created by revising the 10-item Mathematics Anxiety Scale (MAS) developed by Betz (1978) by replacing mathematics terms with statistical terms (Pretorius and Norman, 1992). The internal consistency coefficient for the sample in which the unidimensional SAS-10 scale was developed was reported as 0.94 and the split-half reliability were reported as 0.91. The relationship between the SAS-10 and the STAI (State–Trait Anxiety Inventory; Spielberger et al., 1970), whose reliability and validity have been previously proven, was examined and a statistically significant relationship was found between them.

In addition to the developed statistics anxiety scales, there are many statistics anxiety scales adapted to different cultures in the literature. The number of items, the name of the scale and the number of subscales of the adapted scales vary according to the adapted culture. The widespread use of statistics anxiety scales around the world will bring about the differentiation of the conditions of application of the scale. The reliability of a scale is the degree to which it is free from random errors that may occur during its application (Büyüköztürk et al., 2012). The language in which the measurement tool is applied, the length of the test, the objectivity of scoring, the factors related to the instructions of the scale and the factors related to the application conditions will affect the reliability of the sample. In this case, fluctuations in the reliability values in the samples to which the scale is applied are expected. The situation of fluctuations in the reliability coefficient has brought about the need to investigate this variability in a systematic way. The most accurate way to estimate the reliability coefficient for a scale is to systematically combine the reliability coefficients of the studies in which the scale is used (Şen and Yıldırım, 2023). This method is called “reliability generalization” in the literature. The definition of reliability generalization was first used by Vacha-Haase (1998). According to Vacha-Haase (1998), reliability generalization meta-analysis provides important evidence about the amount and source of variation in reliability value. It provides guidance on whether the scale will be appropriate for the sample to which it will be applied (Taylor, 2012). It also provides important evidence for researchers in interpreting data and comparing results (Leech et al., 2011).

Looking at the literature, it was seen that in addition to the studies examining the level of statistical anxiety, the predictors of statistical anxiety were also addressed (Faber and Drexler, 2019; O'Bryant, 2017; Valle et al., 2021; Zhang et al., 2021). There are also studies investigating the relationship of statistics anxiety with affective factors such as perception, attitude, and self-identity (Altun et al., 2021; Kesici et al., 2011; Mji, 2009; Perepiczka et al., 2011; Sesé et al., 2015). Moreover, studies examining the effect of statistics anxiety on variables such as gender, age, and academic performance were also found (Eshet et al., 2022; Hsiao and Chiang, 2011; Sandoz et al., 2017; Valle et al., 2021; Wu et al., 2022). Apart from the studies investigating the variables associated with statistical anxiety, studies in which reliability generalization meta-analysis was conducted were also examined. It was examined whether the sample type (Özdemir et al., 2020; Shields and Caruso, 2003; Wallace and Wheeler, 2002), the type of publication of the study (Ock et al., 2021), the language in which the measurement tool was applied (Kıyıcı and Kahraman, 2022), and the length of the test (Fitzgerald, 1996), which are thought to affect the mean reliability value obtained, affect the mean reliability value. In addition, reliability generalization meta-analysis studies in which continuous variables such as the year the study was published, the mean score of the scale and the mean age of the sample were investigated as predictors of the mean reliability value are also available in the literature (Sen, 2022; Wallace and Wheeler, 2002). There is no study examining the reliability generalization meta-analysis method on statistics anxiety. Increasing the number of such studies is extremely important in terms of identifying previously examined studies in the literature on reliability generalization meta-analysis and reaching more comprehensive results. It is thought that finding a general reliability value of the statistics anxiety scales to be obtained from the current study will contribute to the relevant literature. Moreover, it is thought that it will guide scientists in future studies. In this direction, while analyzing the studies included in the research, answers to the following questions were sought:

1. What is the reliability induction rate of the studies reached during the research process?
2. What is the mean value of the internal consistency coefficients of each statistics anxiety scale and its sub-dimensions included in the study?
3. What is the mean value of the overall internal consistency coefficients of all statistics anxiety scales included in the study?
4. Do the variables such as the measurement tool, type of publication, continent and education level where the scale is applied, and the language used in the studies included in the research have an effect on the mean value of internal consistency coefficient?
5. Is the mean age of the individuals participating in the study, the ratio of the number of women to the number of men, the mean score of the scale, the standard deviation of the mean score, the year of the study, or the number of items predictive of the mean value of internal consistency coefficients?

## 2 Method

In the current study, reliability generalization meta-analysis method, which is one of the meta-analysis methods, was used by bringing together the reliability coefficients of the studies in which statistics anxiety scales were used. The way to estimate an accurate reliability coefficient for a specific measurement tool is to combine the reliability coefficients obtained from numerous studies (Şen and Yıldırım, 2023, p. 318). The method of combining the results of numerous studies in a meta-analysis to obtain a single result is also used in combining the reliability coefficients of measurement tools. Reliability generalization is a method of estimating a common reliability coefficient by combining the reliability coefficients obtained from a scale used in various studies (Vacha-Haase, 1998, p. 12). In this context, the reliability coefficients in the studies in which the STARS, SAS, WAESTA, SAS-10 scales were used among the statistics anxiety scales were brought together and the reliability generalization meta-analysis method was used for each scale. Moreover, reliability generalization meta-analysis was also performed for the subscales of multidimensional and widely used statistics anxiety scales (STARS, SAS). Furthermore, pooled reliability coefficient was obtained for the whole of the statistics anxiety scales (STARS, SAS, WAESTA, SAS-10, SAM, SAQ). When the reliability coefficient preferred by the studies included in the analysis was examined, it was seen that the most commonly used reliability coefficient was Cronbach’s alpha coefficient, one of the internal consistency coefficients. For this reason, the analyses were conducted with the alpha coefficient.

### 2.1 Data sources and search strategies

**Figure 1:**
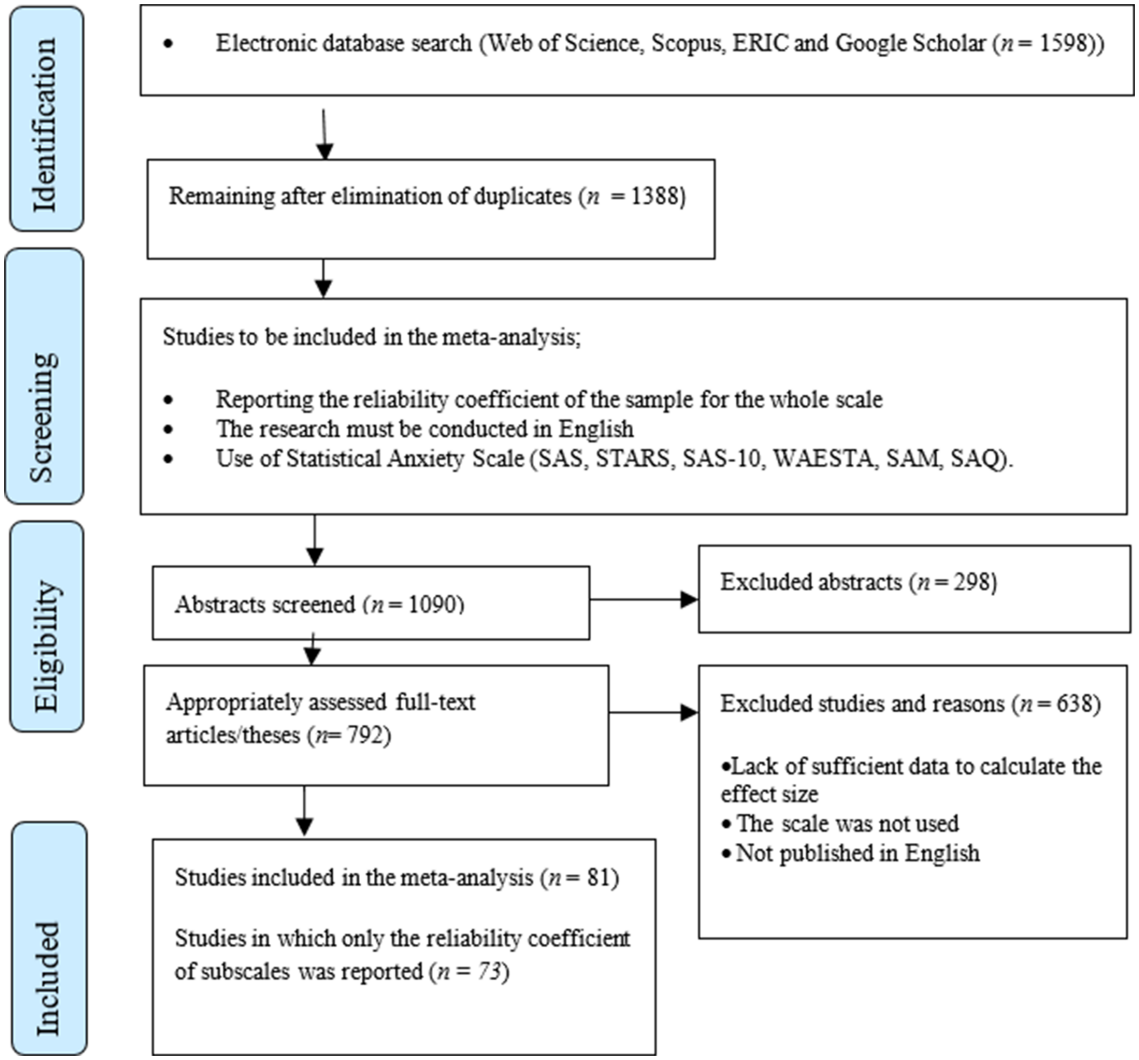
PRISMA flowchart: Studies included in the study.

While reviewing the studies, it was aimed to reach all scales measuring statistics anxiety and all studies in which these scales were used without determining a specific year interval. At this context, Web of Science, Scopus and ERIC databases and Google Scholar search engine were searched, respectively. In the inclusion of the studies in the current study, the criteria of being published in English, using a measurement tool that measures statistics anxiety, and reporting the reliability coefficient of the whole scale or its subscales were taken into consideration. In line with these criteria, databases were searched with some keywords as well as the citations of the statistics anxiety scales identified between May 2023 and July 2023. While ERIC, Web of Science and Scopus were searched with the words “statistical anxiety” and “statistics anxiety,” Google Scholar was searched with the search model “scale OR measure OR questionnaire OR inventory” AND “intitle:statistical anxiety” AND “scale OR measure OR questionnaire OR inventory” AND “intitle:statistics anxiety.” As a result of these searches, a total of 1,598 articles, theses or published papers were reached. Meta-analysis was conducted with 84 alpha coefficients obtained from 81 studies as a result of eliminating 1,444 studies that did not meet the above criteria. Furthermore, 73 studies in which only the reliability coefficient of the subscales was reported were also recorded to be used in the reliability generalization meta-analysis of the subscales. In the selection of studies to be included in the meta-analysis, the process should be carried out in accordance with a checklist. In addition to the REGEMA (Reliability Generalization Meta-Analysis) checklist developed by Sanchez‐Meca et al. (2013) for reliability generalization meta-analysis, PRISMA (Preferred Reporting Items for Systematic reviews and Meta-Analysis) developed by Moher et al. (2009) is the most widely used checklist for meta-analysis. The studies included in the analysis are shown in the PRISMA flow diagram in Figure 1.

The 157 reliability coefficients obtained from 154 studies recorded during the data collection phase were reported. Of the 115 studies in which STARS was used, 24 studies reported the alpha coefficient for the whole scale but not for the subscales. And, there are 27 studies in which the alpha coefficient was reported for the whole STARS and subscales. On the other hand, there are 64 studies in which the alpha coefficient is not reported for the whole STARS, but only for the subscales. A similar situation also applies to SAS. Alpha coefficient for the whole SAS was reported in 5 out of 27 studies in which SAS was used. There are 14 studies reporting alpha coefficient for the whole SAS and subscales. On the other hand, there were 8 studies in which the alpha coefficient was reported for subscales but not for the whole SAS, and 5 studies in which the WAESTA scale was used. Four studies using the SAS-10 scale were recorded. One study using SAM and one study using SAQ were also included in the analysis (Earp, 2007; Idika and Ojong, 2020). In addition, studies using two different statistics anxiety scales (Altun et al., 2021; Chew et al., 2018; Grajzel, 2019; Igbokwe et al., 2017) were recorded as a second study with the same name.

In a study using the SAI recorded during the data collection phase (Zeidner, 1991), only the alpha coefficient for the subscales was reported. Similarly, in a study using the SAM (Vahedi et al., 2011), only the alpha coefficient for the subscales was reported. On the other hand, in two studies (Asare, 2023; Jones et al., 2022) where the reliability coefficient for the subscales of STARS was reported, the omega coefficient was reported. Since only studies reporting alpha coefficients were included in the analysis, the two studies reporting omega coefficients (Asare, 2023; Jones et al., 2022) were excluded from the analysis. In this case, a total of four studies were excluded from the analysis.

### 2.2 Data coding and coder reliability

In addition to the alpha coefficients of the studies included in the analysis, the year of publication, number of items, the scale used, the language in which the scale was applied, the type of publication of the study, the type of sample, the continent in which the scale was applied, were also recorded. Sample size, the ratio of the number of women to the number of men in the sample, the mean scale score, the standard deviation of the mean scale score, and the mean age were also collected. The scales used in these data were coded as STARS, SAS, WAESTA, SAS-10, SAM and SAQ. Moreover, the language in which the scale was administered was coded as English and non-English. The continent variable was coded as Asia, Europe, America, Africa and Australia. But there are studies that collected data from more than one continent (Chew et al., 2018; Gibeau et al., 2023). The sample type variable was coded as undergraduate, graduate and mixed (undergraduate and graduate).

For coder reliability, 20% of the researches were randomly selected from the data. After checking the reliability coefficient and *n* values for the selected data, When the data obtained from the researchers was evaluated, it was found that the inter-researcher agreement coefficient was 100%.

### 2.3 Data analysis

The present meta-analysis study aims to apply a reliability generalization meta-analysis to the 84 reliability coefficients reported for the scales in 81 studies that used different statistics anxiety scales. Additionally, 73 studies reporting reliability coefficients for subscales were included in the study to calculate the mean alpha coefficient for subscales. Analyses were conducted with the CMA v3 program. Method of moments (MM, also known as the DerSimonian and Laird method) was used as estimator method during the analysis.

In the study, firstly, the reliability induction rate was determined. In this way, the case of reporting reliability coefficient in primary studies will be revealed. Since the alpha coefficients of the scales are mostly reported at values of 0.70 and above, the distribution of alpha coefficients will show negative skewness (Beretvas and Pastor, 2003). In this context, the researchers contend that it would be more appropriate to apply a transformation formula to the alpha coefficients (Bonett, 2002, 2010; Hakstian et al., 1976). It was deemed appropriate to apply Bonett (2002) transformation formula to the alpha coefficients in order to stabilize the variance as well as to eliminate the skewness in the alpha coefficients. The transformation formulas for variance variability and alpha coefficients are as follows (Equations 1,2):


$$L_{i}=\ln(1-|{\hat{\alpha }}_{i}|)$$ (1)

$$V(L_{i})=\frac{2J_{i}}{\left(J_{i}-1)(n_{i}-2\right)}$$ (2)

After applying the transformation formula to the alpha coefficients, the effect size to be used in the meta-analysis should be selected. Since the random effects model is mostly used in social sciences and it is thought that there is more than one source of variability in this model, the random effects model was chosen in this study. Following the selection of the effect size, in addition to the forest plot presented to test the heterogeneity of the data group, the *Q-statistic* and the *I^2^* value to determine the amount of heterogeneity were calculated. The Q-statistic aims to test whether there is a statistically significant chi-square value (Sedgwick, 2015). On the other hand, 50% percentile value is a limit for the calculated *I^2^* value (Cleophas and Zwinderman, 2007). After the heterogeneity of the data group was tested, analog to the ANOVA analysis was conducted under the mixed effects model in the analysis of categorical variables in order to determine the sources of heterogeneity (Hedges, 1982). In the analysis of continuous variables, meta-regression analysis with maximum likelihood estimation method was applied (Hedges and Olkin, 2014). Furthermore, the *Q_B_* -statistic (Q-between) was used to determine statistical significance in moderator variables. R^2^ estimation was used to determine the proportion of variance explained by continuous variables in meta-regression analysis (Yıldırım, 2021).

In the study, six different methods are mentioned to determine whether there is publication bias (Card, 2015). These methods are the funnel plot (Light and Pillemer, 1984), the trim and fill method developed by Duval and Tweedie (2000), the fail-safe *N* method proposed by Rosenthal (1979) and Orwin (1983) fail-safe *N* method. Analyses were performed with these methods. In addition, the regression analysis developed by Egger et al. (1997) and the rank correlation test developed by Begg and Mazumdar (1994) were utilized. The data was analyzed using the CMA v3 program.

### 2.4 Demographic characteristics of the primary studies

**Figure 2:**
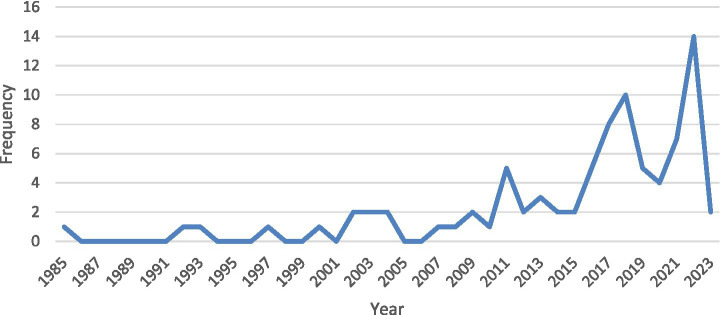
Number of studies using statistics anxiety scale according to years.

The distribution of the studies in which statistics anxiety scales were used according to years is shown in Figure 2. When Figure 2 is examined, it is seen that the use of statistics anxiety scales has increased over the years and the most commonly used year is 2022.

The demographic characteristics of the studies included in the analysis are shown in Table 1. When Table 1 is examined, the measurement tools consist of 6 different scales, namely STARS (61.90%), SAS (25.00%), WAESTA (5.95%), SAS-10 (4.76%), SAM (1.19%) and SAQ (1.19%). When the publication types of the studies were analyzed, it was seen that they consist of thesis (13.04%), article (76.19%) or paper (10.71%). The measurement tools were applied not only in the continent where they were developed but also in Asia (19.04%), Europe (29.76%), America (39.28%), Africa (11.90%) and Australia (1.19%). The educational level of the participants was grouped in three different levels: undergraduate students (68.24%), graduate students (23.52%), and mixed (8.24%). The studies using the instruments measuring statistics anxiety were grouped in two different ways: studies using the English version (56.52%) and studies using non-English versions (43.47%). The non-English versions were Arabic, Malay, Italian, German, Turkish, Chinese, Spanish, Hebrew, French and Dutch. Eighty-four reliability coefficient obtained from 81 studies for reliability generalization meta-analysis are presented in Supplementary Table 1. As stated in Supplementary Table 1, no year range was determined during the screening, and it was aimed to reach all published studies. In Supplementary Table 1, in addition to the reliability coefficients of the samples, the number of the sample, the ratio of the number of women to the number of men in the sample, the scale applied, the number of items in the scale, the continent where the study was conducted, the type of study published, the education level of the participants, and the language in which the scale was applied are given. There is also information on the number of items of the measurement tool, the year of publication of the study and the mean age of the sample.

**Table 1:** Characteristics of the studies included in the study.

	Frequency	%
Measurement tool (*n* = 84)		
STARS	52	61.90
SAS	21	25.00
WAESTA	5	5.95
SAS-10	4	4.76
SAM	1	1.19
SAQ	1	1.19
Type of publication (*n* = 84)		
Article	64	76.19
Thesis	11	13.04
Proceeding paper	9	10.71
Continent (*n* = 84)		
Asia	16	19.04
Europe	25	29.76
America	33	39.28
Africa	10	11.90
Australia	1	1.19
Education level (*n* = 84)		
Postgraduate	20	23.80
Undergraduate	57	67.85
Mixed	7	8.33
Applied language of the scale (*n = 69*)		
English	39	56.52
Non-English	30	43.47

According to Supplementary Table 1, there were a total of 18,809 participants in the studies included in the meta-analysis. In addition to the number of participants, there are also studies reporting the mean age of participants. In 53 of the 81 studies, the mean age of the participants was reported. According to the reported mean ages, the mean age of the participants ranged between 18.23 and 40.73. The number of studies reporting the gender of the participants is also quite high. Only 19 studies did not report the number of men and women participating in the study, while the number of men and women was reported in the remaining 65 studies. Accordingly, in the current study, the ratio of the number of women participating in the research to the number of men was taken for each study. The female to male ratio of the participants is between 0.70 and 12.67.

Since the number of items of the measurement tools used in the studies is also an important variable for reliability generalization meta-analysis, the number of items for each study is reported. The number of items of the scales varied between 10 and 51. In addition, there are 21 studies reporting the mean scale score recorded during the data collection phase. The mean scale scores of these studies ranged from 43.46 to 198.84 for STARS and from 46.10 to 74.70 for SAS. For WAESTA, the mean scores ranged from 40.62 to 42.66, and for SAS-10, the mean scale scores ranged from 26.07 to 34.19. The standard deviation of the mean scale scores of these studies is between 1.1 and 59.06. The publication years of the studies included in the analysis ranged between 1985 and 2023 (*Median* = 2017).

**Figure 3:**
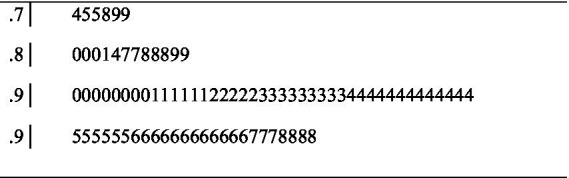
Stem and leaf plot of raw alpha values.

Figure 3 shows the stem and leaf graphs of the 84 raw alpha values. When the alpha coefficients are analyzed, it can be inferred that the reliability of all of these values is at a sufficient level (O’Rourke et al., 2005). In Figure 3, the unweighted mean alpha coefficient was calculated as 0.913 (*Median* = 0.930, *SD* = 0.056). According to the graph, the lowest alpha coefficient was 0.74 and the highest alpha coefficient was 0.98. The values of the 84 reliability coefficients show an asymmetry of −1.514.

## 3 Results

### 3.1 Reliability induction

In some studies, instead of reporting the reliability coefficient of the sample, the reliability coefficient of the sample in which the scale was developed is reported. This situation is called reliability induction in the literature (Shields and Caruso, 2004; Şen and Yıldırım, 2023). In the current research, there are quite a number of studies in which the reliability coefficients of the sample of the studies in which statistics anxiety scales were used were not reported. In 204 studies in which statistics anxiety scales were used, the reliability coefficient of the applied sample was not reported and was not included in the analysis. In this case, 157 studies in which the reliability coefficient of the sample to which the scale was applied was reported were included in the analysis. Since 204 studies in which the statistics anxiety scale was used and the reliability coefficient was not reported were not included in the analysis, the reliability induction rate was determined as 57.2%. As can be seen from here, more than half of the studies that should have used the measurement tool and reported reliability did not take this requirement into account. This can be considered as an important methodological problem.

### 3.2 Publication bias

**Figure 4:**
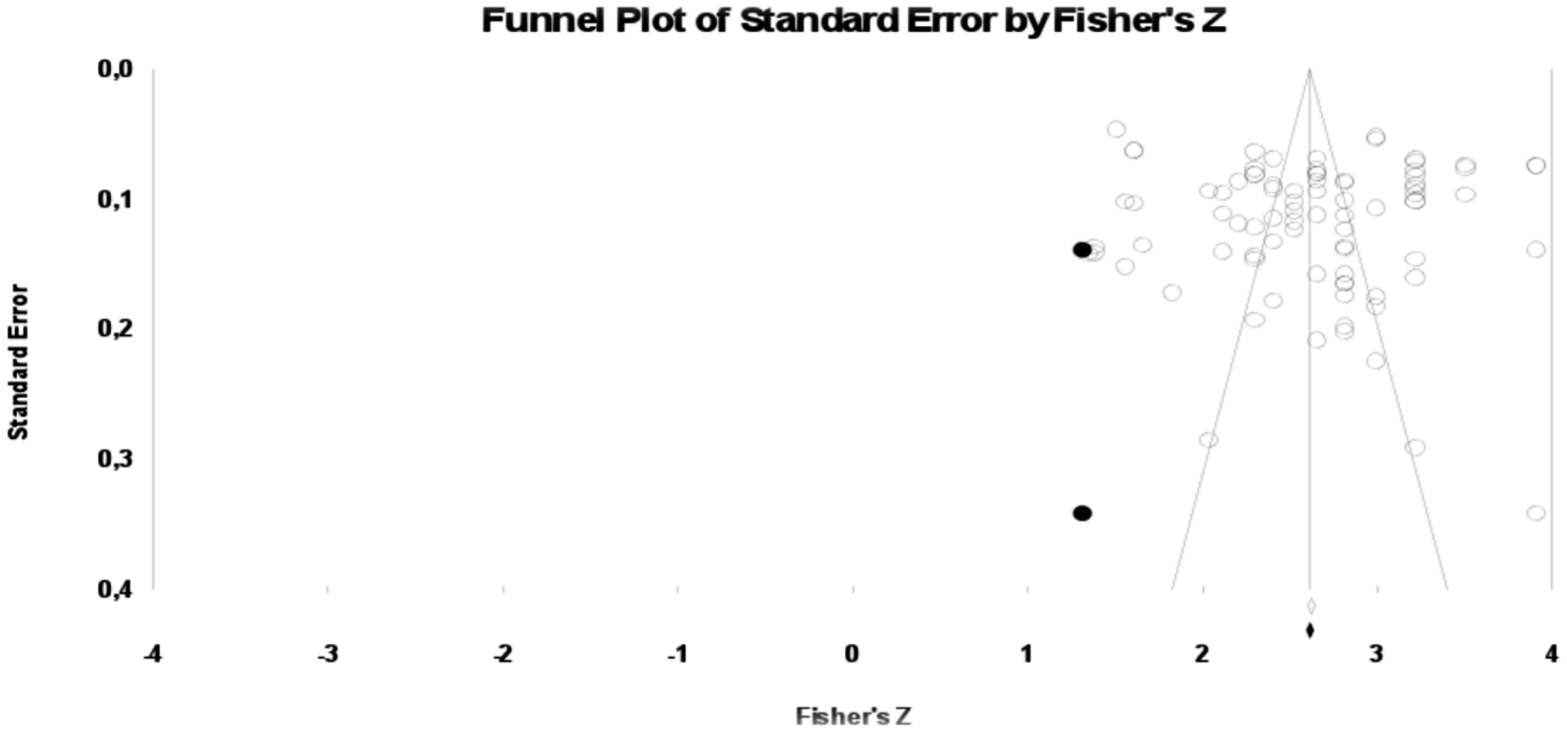
Funnel plot obtained as a result of the trim and fill method.

In order to determine whether there was any publication bias in the data group. According to the classical fail-safe *N* value, the value was found to be 10,026. This value is considerably higher than the value of 425 obtained with the formula *N_R_* (5 k + 10). In this case, it is seen that the combined alpha coefficients are not biased according to the classical perpetrator-safe *N* method. According to Orwin’s fail-safe *N* method, the number of missing data should be 2,120 for Fisher’s *z* to be 0.01. Since this number is almost impossible to reach, it can be said that the current study is not biased according to Orwin’s fail-safe *N* method. Moreover, according to the findings of Begg and Mazumdar’s rank correlation test, Kendall’s two-tailed *p* value is not statistically significant (*p = 0*.432) and the standard error value is negative (*τ* = −0.05). According to this test, the two-tailed *p* value should not be statistically significant to ensure the absence of publication bias. In addition, according to the Egger’s test analyzed, the *t* value is not statistically significant [*t*_(83)_ = 0.183, *p* = 0.427]. Egger’s one-tailed *p* > 0.05 indicates that the current finding is not biased. Finally, the findings of Duval and Tweedie’s trim and fill method are shown in Figure 4.

**Figure 5:**
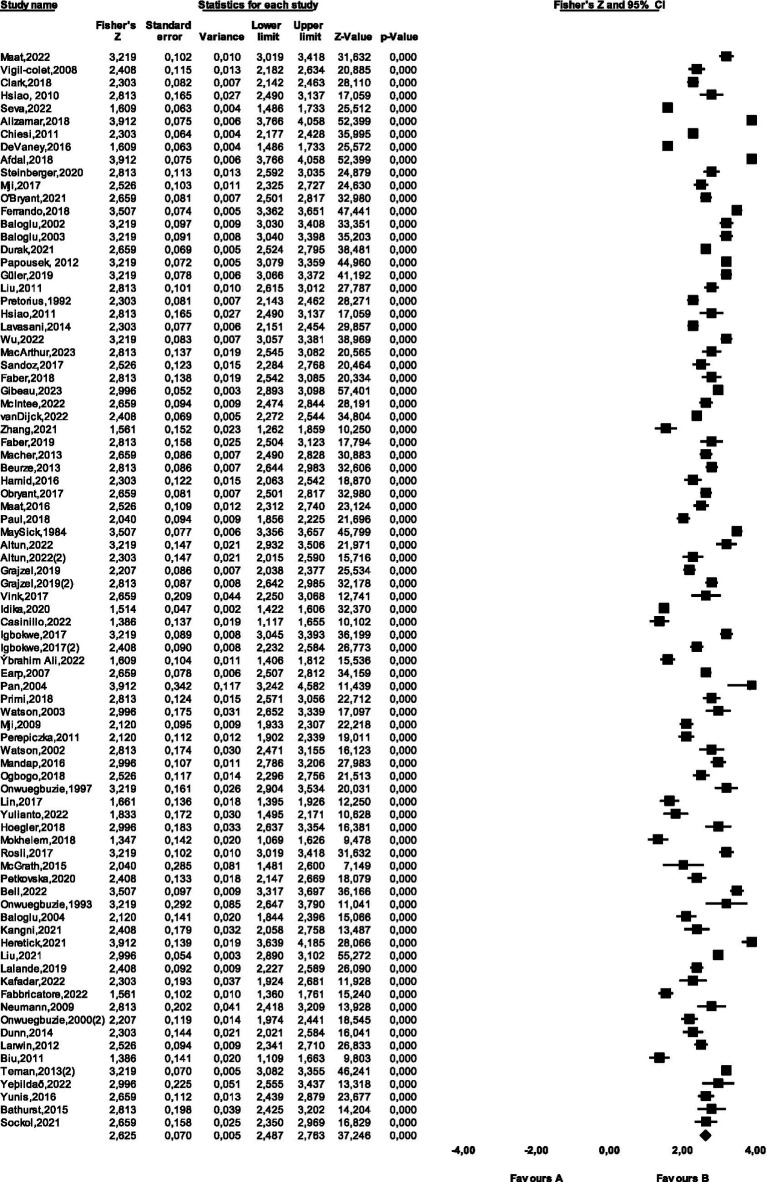
Forest plot of the studies included in the meta-analysis.

When Figure 4 is examined, the data shows a symmetrical distribution. In addition, the fact that there are only two fictitious studies that need to be added to ensure that the publication is not biased shows that the data group is not biased. Moreover, the forest plots created to determine the heterogeneity of the data group is shown in Figure 5.

### 3.3 Reliability generalization meta-analysis for each scale

The statistics anxiety scales included in the reliability generalization meta-analysis were analyzed with a total of six different measurement tools: STARS, SAS, SAS-10, WAESTA, SAQ and SAM. It was aimed to conduct reliability generalization meta-analysis for each of these scales, and since only one study using SAM and SAQ scales was included in the analysis, no reliability generalization meta-analysis was conducted for these studies. Accordingly, the reliability coefficients of the studies using the STARS, SAS, SAS-10 and WAESTA scales as well as the pooled reliability values of the subscales of STARS and SAS are shown in Table 2. Table 2 also includes the descriptive statistics of these measurement tools.

**Table 2:** Mean reliability values of statistics anxiety scales and subscales.

Measurement tool	Sub scales	Rp. α	*k*	*n* of items	Mean α	*95%CI*	[min, max]	*Q*(sd)	*I^2^*	Orwin *N*
STARS		0.96	52	51	0.931	[0.917, 0.942]	[0.740, 0.980]	2016.83(51)*	97	1,389
	WS	0.94	85	16	0.911	[0.909, 0.912]	[0.710, 0.960]	1810.84(84)*	95	1968
	IA	0.87	90	11	0.864	[0.851, 0.871]	[0.640, 0.950]	1578.94(89)*	94	1,691
	TCA	0.68	91	8	0.865	[0.854, 0.877]	[0.680, 0.950]	1348.24(90)*	93	1719
	CSC	0.88	83	7	0.847	[0.831, 0.861]	[0.640, 0.960]	1611.61(82)*	95	1,514
	FAH	0.89	88	4	0.822	[0.805, 0.837]	[0.600, 0.930]	1469.51(87)*	94	1,446
	FST	0.80	82	5	0.780	[0.766, 0.793]	[0.580, 0.870]	618.47(81)*	87	1,189
SAS		0.91	21	24	0.917	[0.898, 0.933]	[0.790, 0.950]	713.10(20)*	97	517
	EA	0.87	22	8	0.895	[0.911, 0.940]	[0.780, 0.960]	570.84(21)*	96	477
	AHA	0.92	22	8	0.927	[0.850, 0.898]	[0.840, 0.980]	774.37(21)*	97	547
	IA	0.82	21	8	0.876	[0.876, 0.911]	[0.730, 0.920]	662.21(20)*	97	435
WAESTA		0.94	5	17	0.951	[0.941, 0.960]	[0.940, 0.960]	10.73(4)*	63	150
SAS-10		0.90	4	10	0.918	[0.836, 0.959]	[0.750, 0.980]	70.14(3)*	96	85

WS = Worth of Statistics; IP = Interpretation anxiety; TCA = Test and Class Anxiety; CSC = Computational Self-Concept; FAH = Fear of Asking Help; FST = Fear of Statistics Teacher; EA = Examination Anxiety; AHA = Asking for Help Anxiety; IA = Interpretation Help; k = number of studies; Rp.α = original alpha at the scale development stage. *p < 0.01.

The internal consistency coefficient of the sample in which the measurement tools were developed, the total number of studies in which the scale was used and the number of items in the scale are shown in Table 2. Moreover, for each of these scales, the 95% confidence interval of the alpha values for which Bonett (2002) transformation formula was applied, and the lowest and highest alpha coefficients in the included studies were also presented. In addition, the *Q* statistic with degrees of freedom for testing heterogeneity and *I^2^* values for the amount of heterogeneity are shown. Finally, Orwin’s fail-safe *N* value was examined to determine whether the data formed by the alpha coefficients of the scales were subject to publication bias.

According to Table 2, the alpha coefficient of the sample in which STARS was developed was reported as 0.96, while the original alpha coefficients of the subscales were 0.94, 0.87, 0.68, 0.88, 0.89 and 0.80, respectively. In addition to the 52 studies in which STARS was used and included in the analysis, the number of studies in which subscales were used and included in the analysis were 85, 90, 91, 83, 88 and 82, respectively. As a result of the reliability generalization meta-analysis applied to the alpha coefficients of these studies with a range between 0.740 and 0.980, the mean alpha coefficient for the whole STARS was calculated as 0.931 (95% CI: 0.917–0.942), and the mean alpha coefficients for the subscales were calculated as 0.911, 0.864, 0.865, 0.847, 0.822 and 0.780, respectively. Table 2 shows that heterogeneity was achieved according to the *Q* values calculated for the whole STARS and each of the subscales (*p* < 0.01). Furthermore, the *I^2^* values calculated for the heterogeneity amounts of these scales are quite high (>75%). Considering the Orwin fail-safe *N* value examined for publication bias, it can be concluded that the data groups formed for STARS and subscales of STARS are not biased.

Table 2 presents the findings of the reliability generalization meta-analysis for the whole 24-item SAS and its three subscales as well as demographic characteristics. While the alpha coefficient for the sample in which the SAS was developed was reported as 0.91, the original alpha coefficients for the subscales of the SAS were 0.87, 0.92 and 0.82, respectively. In addition to reporting the alpha coefficients of 21 studies in which the SAS was used, the number of studies in which the alpha coefficients of the subscales were also reported were 22, 22 and 21, respectively. As a result of the reliability generalization meta-analysis applied to alpha values ranging from 0.790 to 0.950, the mean alpha value for the whole SAS was determined as 0.917 (95% CI: 0.989–0.933). The mean alpha coefficients for the subscales of the SAS were 0.895, 0.927 and 0.876, respectively. According to the *Q* value calculated for the whole and subscales of the SAS, heterogeneity was achieved (*p* < 0.01) and the *I^2^* value calculated for the amount of heterogeneity was quite high (>75%). Considering the Orwin fail-safe *N* value examined for publication bias, it can be concluded that the data groups created for the SAS and its subscales are not biased.

Although the 17-item WAESTA scale has three subscales, reliability generalization meta-analysis was conducted for this scale since there are few studies using the WAESTA scale included in the analysis in the current study. Reliability generalization meta-analysis was not applied to the subscales. Five studies using this scale were included in the analysis and the alpha coefficient for the sample in which WAESTA was developed was reported as 0.94. The mean alpha coefficient of the studies using the WAESTA scale with a range between 0.940 and 0.960 was analyzed as 0.951 (95% CI: 0.941–0.960). According to the Q-statistic calculated to test the heterogeneity of the studies using WAESTA, heterogeneity was achieved (*p* < 0.01). In addition, the *I^2^* value calculated for the amount of heterogeneity exceeded the 50% limit. Considering the Orwin fail-safe *N* value examined for publication bias, it can be concluded that the data groups created for WAESTA are not biased.

Four studies using the 10-item, unidimensional SAS-10 scale were included in the analysis and the range of these studies varied between 0.750 and 0.980. The alpha coefficient of the sample in which SAS-10 was developed was reported as 0.90. The mean alpha coefficient of the studies using this scale was analyzed as 0.918 (95% CI: 0.836–0.959). The heterogeneity of the analyzed data group was ensured according to the calculated *Q-statistic* (*p* < 0.01) and the amount of heterogeneity was quite high (>75%) according to the calculated *I^2^* value. Considering the Orwin fail-safe *N* value examined for publication bias, it can be concluded that the data groups created for SAS-10 are not biased.

### 3.4 Overall effect size

In addition to the reliability generalization meta-analysis conducted for each of the scales, all of the studies using these scales were combined and the overall alpha mean of all studies was created. In Table 3, the general reliability coefficient formed by combining the reliability coefficients of the studies included in the meta-analysis is reported under the random effects model. Moreover, the heterogeneity test for the model is also presented in Table 3. The *Q* value calculated to determine the heterogeneity of the data group was reported as 3596.36. This value is well above the 0.05 confidence interval limit (*sd* = 83, χ2 = 105.27) with 84 degrees of freedom in the chi-square table. In this case, the heterogeneity of the data is ensured. The *I^2^* value calculated to determine the amount of heterogeneity was found to be 97.6%. This value shows that the amount of heterogeneity of the data group is quite high.

**Table 3:** Effect sizes and heterogeneity test.

Model	*k*	Mean α	SE	*Z*	p	95% CI	*df*	*Q*	*p*	*I* ^2^
Random effects	84	0.928	0.07	37.53	0.00	[0.917, 0.937]	83	3593.76	0.00	97. 69

k = number of studies, df = degrees of freedom, CI = confidence interval.

The mean of the alpha coefficients using Bonett (2002) transformation formula was 0.928 (95% CI: 0.917–0.937) under the random effects model and this value is statistically significant (*p* < 0.01). It is seen that the mean of the transformed alpha values is higher than the mean of the raw alpha values shown in Figure 3.

### 3.5 Sub-group analysis of categorical variables

In addition to the statistical significance of all studies included in the analysis, categorical and continuous variables were determined to investigate the source of heterogeneity of the studies. Analog to the ANOVA analysis was performed for categorical variables and meta-regression analysis for continuous variables. In Supplementary Table 1, five different categorical variables were identified: the type of scale used, the type of publication of the study, the language in which the scale was applied, the educational level of the sample, and the continent in which the study was applied. Moreover, the continuous variables in Supplementary Table 1 are the year of publication of the study, the ratio of the number of women to the number of men in the sample, the number of items in the scale, the sample size, the mean age of the sample, the mean score of the scale and the standard deviation of the mean score of the scale. Analog to the ANOVA findings for categorical variables are shown in Table 4.

**Table 4:** Analog to the ANOVA results of categorical variables.

Variable	Category	*k*	Mean α	*95%CI*	*Q_B_*	*df*	*p*
Scale	STARS	52	0.931	[0.917, 0.943]	**14.364**	**3**	0.002*
	SAS	21	0.917	[0.898, 0.933]			
	WAESTA	5	0.952	[0.941, 0.960]			
	SAS-10	4	0.918	[0.836, 0.960]			
Education level	Undergraduate	57	0.923	[0.908, 0.935]	2.656	2	0.276
	Graduate	19	0.939	[0.924, 0.951]			
	Mixed	8	0.927	[0.900, 0.947]			
Continent	Africa	9	0.899	[0.847, 0.933]	2.780	3	0.427
	America	33	0.931	[0.916, 0.944]			
	Asia	16	0.927	[0.897, 0.948]			
	Europa	24	0.930	[0.910, 0.945]			
Language	English	40	0.929	[0.914, 0.941]	0.619	1	0.431
	Non-English	32	0.937	[0.922, 0.948]			
Publication type	Article	64	0.924	[0.912, 0.934]	1.142	2	0.565
	Dissertation	11	0.933	[0.905, 0.953]			
	Proceeding papers	9	0.939	[0.899, 0.963]			

k = number of alpha coefficients, df = degree of freedom.*p <. 05.

In the analog to the ANOVA analysis, the difference between the scales was first investigated. STARS (*k* = 52), SAS (*k* = 21), WAESTA (*k* = 5) and SAS-10 (*k* = 4) scales were analyzed with alpha coefficients. Since there was only one study using SAM and SAQ scales, they were not included in the analog to the ANOVA analysis. It was observed that the mean alpha coefficients of the 83 studies included in the analog to the ANOVA analysis differed according to the scale types (*Q* = 14.364, *sd* = 3, *p* < 0.05). In this case, it can be said that the heterogeneity of the mean alpha coefficient is due to the type of scale used. When the mean alpha coefficients of the scale types are compared, it is seen that the scale with the highest mean alpha value is WAESTA (0.952), followed by STARS (0.931), and finally SAS (0.918) and SAS-10 (0.918) scales together.

No statistically significant difference was found between the mean alpha coefficients of other categorical variables in the current study (*p* > 0.05). According to Table 4, no statistically significant difference was found according to the variables of education level (*Q* = 2.656, *sd* = 2, *p* = 0.265), continent where the scale was applied (*Q* = 2.780, *sd* = 3, *p* = 0.433), language where the scale was applied (*Q* = 0.619, *sd* = 1, *p* = 0.431) and type of publication of the study (*Q* = 1.142, *sd* = 2, *p* = 0.613). The non-significant *p* values indicate that comparable findings were not reached between subgroups.

### 3.6 Moderator analysis of continuous variables

In the present study, continuous variables were determined in addition to categorical variables. The year the study was published, the size of the sample, the number of items in the scale, the mean age of the sample, the ratio of the number of women to the number of men in the sample, the mean scale score and the standard deviation of the mean scale score were determined as continuous variables. The findings of the meta-regression analysis applied to investigate the effect of each continuous variable on the mean alpha coefficient are shown in Table 5. The reason for applying meta-regression analysis separately for each variable instead of multiple is missing data. Since the data of different studies could not be accessed under each heading, there are less than 15 studies when the general model is established.

**Table 5:** Results of meta-regression analysis applied separately for each continuous variable.

Continuous variables	*k*	*ß*	*B*	SE	*p*	*R* ^2^	*Q_E_*
Year	84	30.452	−0.014	0.008	0.101	0.03	3518.67*
Sample size	84	2.631	−0.000	0.000	0.940	0.00	3478.22*
Number of items	84	2.383	0.007	0.004	0.121	0.03	3417.17*
Mean age	52	2.091	0.016	0.015	0.265	0.01	2006.66*
Male/female	65	2.633	0.002	0.030	0.950	0.00	2845.02*
Scale score mean	21	2.150	0.006	0.002	0.012**	0.24	1024.92*
Scale SD	21	2.256	0.020	0.090	0.024**	0.24	627.87*

k = number of alpha coefficients; b_j_ = unstandardized regression coefficients; ß = slope value; B = unstandardized regression coefficients; SE = standard error; R^2^ = explained variance; Q_E_ = Q value for residual values; *p < 0.01; **p <. 05.

According to the meta-regression findings, the variables of scale mean score and standard deviation of scale mean score are statistically significant predictors of the mean reliability value (*p* < 0.05). As predicted, there is a significant and positive relationship between these variables and mean alpha values. A one-unit increase in the mean scale score is expected to increase the mean reliability value by 0.0056. Furthermore, a one-unit increase in the standard deviation of the mean scale score is expected to increase the mean reliability value by 0.0198 points. Here, it is seen that the standard deviation of the mean scale score affects the mean alpha value more than the mean scale score. The *R*^2^ value, which is the indicator of the explained variance ratio, is 24% for the mean scale score and 24% for the standard deviation of the mean scale score. According to Table 5, other continuous variables were not statistically significant predictors of the mean alpha coefficient (*p* > 0.05). In the multiple regression model for the combined reliability value, the entire model was statistically significant (*Q_M_* = 3593.76, *p* < 0.01) and explained 98% of the total variance. In addition, the model for residual values is also statistically significant (*Q* = 3424.61, *p* < 0.01).

## 4 Conclusion and discussion

The aim of the present study is to establish an overall reliability value for all of the statistics anxiety scales, as well as to apply reliability generalization meta-analysis for each statistics anxiety scale (STARS, SAS, WAESTA, SAS-10). Bonett (2010) conversion formula was applied to the alpha coefficients of the studies in which statistics anxiety scales were used. Since the mean alpha value obtained from the transformed alpha coefficients is above 0.90, the applications are reliable (Büyüköztürk, 2021). In the literature, there are studies in which the mean alpha coefficient of each scale was calculated in addition to reliability generalization meta-analysis for different scales measuring the same construct (Graham and Christiansen, 2009; Graham et al., 2011). Reliability generalization meta-analysis was also conducted for the subscales of multidimensional statistics anxiety scales. As a result of this analysis, it was concluded that the reliability of each subscale was above 0.70 (Clark and Watson, 2016). When the literature is examined, there are studies in which the mean alpha coefficients for the subscales were analyzed in addition to the mean alpha value for the entire measurement tool (Kıyıcı and Kahraman, 2022; Graham and Christiansen, 2009). This is similar to the method of the current study. In addition to the reliability generalization meta-analysis applied for each scale, reliability generalization meta-analysis was also applied for all of the statistics anxiety scales. Since the mean alpha coefficient obtained as a result of the analysis conducted under the random effects model was above 0.70, it was seen that the applications of the scale were reliable (DeVellis, 1991; Tavakol and Dennick, 2011). Moreover, the heterogeneity of the data group was ensured and it was determined that the amount of heterogeneity was quite high. The scope of the current study includes articles, proceedings, and theses that meet the specified criteria and use at least one of the scales mentioned above, accessible via the Web of Science, Scopus, ERIC databases, and Google Scholar search engine as of July 2023. Only studies published in Turkish or English were included in the research. In many studies, it was observed that the reliability coefficient of the sample in which the scale was used was not reported. Since the conditions of each application of the scale are different, the reliability value should be reported (Shields and Caruso, 2003).

In order to investigate the sources of heterogeneity in the heterogeneous data group, analog to the ANOVA was applied for categorical variables and meta-regression analysis was applied for continuous variables. The categorical variables for which analog to the ANOVA was applied were examined in five subgroups: the type of scale, the educational level of the sample, the continent in which the scale was applied, the language in which the scale was applied, and the type of publication of the study. In line with the findings obtained, it is seen that the mean alpha value differs statistically significantly according to the scale type variable. Grajzel (2019) applied STARS and SAS measurement tools to the same sample and reported the alpha coefficient of STARS as 0.890 and the alpha coefficient of SAS as 0.940. This does not coincide with the results obtained in the present study on the other hand, Igbokwe et al. (2017), similar to Grajzel (2019), reported the alpha coefficients of STARS and SAS measurement tools for the same sample as 0.960 for STARS and 0.910 for SAS. This supports the Analog to the ANOVA findings of the current study classified according to scale type.

In the present study, the mean alpha coefficient does not differ statistically significantly according to other categorical variables (the educational level of the sample, the continent where the scale was applied, the language in which the scale was applied, the type of publication of the study). In the literature, there are studies in which statistics anxiety differs according to the educational level of the sample (Fitzgerald, 1996), as well as studies in which there is no statistically significant difference between statistics anxiety and educational level (Benson, 1989). On the other hand, there are studies in the literature where there is a difference in the level of statistics anxiety according to continents (Kesici et al., 2011; Onwuegbuzie, 1999). Moreover, although there was no statistically significant difference in the current study in terms of the language in which the scale was applied, there is a study in the literature in which there was a difference in the level of statistics anxiety according to the language in which the scale was applied (Förster and Maur, 2015). In this study, it was revealed that the group whose mother tongue was different had more statistics anxiety than the group whose mother tongue was German. The reason for this situation can be explained by the inability to understand statistical concepts correctly, followed by a higher level of statistics anxiety. In the present study, no statistically significant difference was found between statistics anxiety and the type of publication of the study. In a different study using reliability generalization meta-analysis method (Sen, 2022), no difference was found between the mean alpha coefficient and the type of publication. On the other hand, there is a study showing that the type of extension is one of the variables affecting statistics anxiety (Fitzgerald, 1996). Since the aforementioned study tried to explain statistics anxiety together with attitude and achievement factors, different results may have been obtained from the present study.

In order to determine the continuous variables affecting the mean alpha coefficient, meta-regression analysis was performed for seven moderator variables (year of publication of the study, size of the sample, number of items, mean age of the sample, ratio of the number of women to the number of men, mean scale score and standard deviation of the mean scale score). As a result of the analysis, it was found that the mean scale score and the standard deviation of the mean scale score were statistically significant predictors of the mean alpha value. This may be due to the different number of items in the scales. Similarly, in another study in which reliability generalization meta-analysis was applied (López-Pina et al., 2015), the mean scale score and standard deviation of the mean scale score were found to be predictors of the mean reliability value. This supports the current study. On the other hand, unlike the conclusion of the current study that year of publication has no effect on statistics anxiety, a study concluding that year of publication has an effect on statistics anxiety was found (Fitzgerald, 1996). Since the year of publication was classified and analyzed as categorical variables in groups of ten instead of continuous variables in the aforementioned study, different results may have been reached. In addition, the result that the mean alpha coefficient was not affected by the sample size in the current study was also reached in a study (Sen, 2022) in which reliability generalization meta-analysis was applied. In the present study, the number of items was not one of the variables affecting the mean alpha coefficient. In the literature, it was found that test length affects statistics anxiety (Fitzgerald, 1996). Contrary, in a study investigating attitude towards mathematics (Bradford, 1990), it was revealed that the length of the test affects attitude. In the aforementioned studies, test length was classified and transformed into a categorical variable and analyzes were conducted in this way. In this case, it can be considered natural to reach different results with the present study. On the other hand, the study concluded that the mean age had no effect on the mean alpha coefficient. Another variable whose effect on the mean alpha coefficient was investigated is the mean age of the sample. In the present study, it was concluded that the mean age did not affect the mean alpha coefficient. Similarly, there are studies in the literature (Bui and Alfaro, 2011; Fitzgerald, 1996) that concluded that age has no effect on statistics anxiety. On the other hand, there are also studies showing that groups with a higher mean age exhibit higher statistics anxiety than younger groups (Bell, 2003; Edirisooriya and Lipscomb, 2021). Since younger groups are more familiar with computerized applications (Prensky, 2001), they may have lower statistics anxiety in statistical applications. Finally, in this study, it was determined that the ratio of the number of women to the number of men in the sample had no effect on the mean alpha coefficient. In the literature, there are studies that show that the level of statistics anxiety differs statistically significantly according to gender, rather than the ratio of the number of women to the number of men in samples that aim to determine statistics anxiety (Edirisooriya and Lipscomb, 2021; Gibeau et al., 2023; Hsiao and Chiang, 2011; MacArthur, 2020). In her study, Demaria-Mitton (1987) found that the level of statistics anxiety did not differ statistically significantly according to gender. Furthermore, Mandap (2016) investigated the difference of statistics anxiety of the group to which STARS was applied according to gender on the subscales of STARS. According to the results, a statistically significant difference was found only for one of the subscales according to gender. According to the findings, women’s level of fear of asking for help was found to be higher than that of men. When we look at this result, it is seen that there is a statistically significant difference only for one of the subscales rather than the whole scale. For this reason, it can be considered that the aforementioned study may have differed from the result of the current study, since analyzes were not conducted for the whole scale.

Bonett (2010) conversion formula was applied to the alpha coefficients in the study. In future studies, different conversion formulas (Fisher *z*, Hakstian et al., 1976) can be applied to different reliability values (composite reliability, test–retest). In addition, researchers who want to examine the variables affecting statistical anxiety, unlike the variables investigated in the current study, can examine the effect of variables such as the field of education of the scale and the country where the scale is applied.

## Data Availability

The original contributions presented in the study are included in the article/supplementary material, further inquiries can be directed to the corresponding author/s.

## References

[ref1] Akdeniz F. (2015). İstatistikte yeni eğilimler ve gelişmeler. Sosyal Bilimler Araştırma Dergisi 4, 1–11.

[ref2] Asare P. Y. (2023). Profiling teacher pedagogical behaviours in plummeting postgraduate students’ anxiety in statistics. Cogent Educ. 10:2222656. doi: 10.1080/2331186X.2023.2222656

[ref3] Begg C. B. Mazumdar M. (1994). Operating characteristics of a rank correlation test for publication bias. Biometrics 50, 1088–1101. doi: 10.2307/2533446, 7786990

[ref4] Bell J. A. (2003). Statistics anxiety: the nontraditional student. Education 124, 157–163.

[ref5] Bendig A. W. Hughes J. B. (1954). Student attitude and achievement in a course in introductory statistics. J. Educ. Psychol. 45, 268–276. doi: 10.1037/h0057391

[ref6] Benson J. (1989). Structural components of statistical test anxiety in adults: an exploratory model. J. Exp. Educ. 57, 247–261. doi: 10.1080/00220973.1989.10806509

[ref7] Beretvas S. N. Pastor D. A. (2003). Using mixed-effects models in reliability generalization studies. Educ. Psychol. Meas. 63, 75–95. doi: 10.1177/0013164402239318

[ref8] Betz N. E. (1978). Prevalence, distribution and correlates of math anxiety in college students. J. Couns. Psychol. 25, 441–448. doi: 10.1037/0022-0167.25.5.441

[ref9] Bonett D. G. (2002). Sample size requirements for testing and estimating coefficient alpha. J. Educ. Behav. Stat. 27, 335–340. doi: 10.3102/10769986027004335

[ref10] Bonett D. G. (2010). Varying coefficient meta-analytic methods for alpha reliability. Psychol. Methods 15, 368–385. doi: 10.1037/a0020142, 20853952

[ref11] Bradford J. W. (1990). A meta-analysis of selected research on student attitudes towards mathematics: The University of Iowa.

[ref12] Büyüköztürk Ş. (2021). Sosyal Bilimler İçin Veri Analizi El Kitabı (29. baskı). Pegem Akademi, Ankara.

[ref13] Büyüköztürk Ş. Kılıç Çakmak E. Akgün Ö. E. Karadeniz Ş. Demirel F. (2012). Örnekleme yöntemleri.

[ref14] Card N. A. (2015). Applied meta-analysis for social science research. New York: Guilford Publications.

[ref15] Clark L. A. Watson D. (2016). Constructing validity: basic issues in objective scale development. Psychological Assessment 7, 309–319. doi: 10.1037/14805-012 PMC6754793 30896212

[ref16] Cleophas T. J. Zwinderman A. H. (2007). Meta-analysis. Circulation 115, 2870–2875. doi: 10.1161/CIRCULATIONAHA.105.594960, 17548743

[ref17] Cruise R. J. Cash R. W. Bolton D. L. (1985). “Development and validation of an instrument to measure statistical anxiety” in Paper presented at the annual meeting of the American Statistical Association statistics education section, Las Vegas, Nevada.

[ref18] Demaria-Mitton P. A. 1987 Locus-of-control. Gender and type of major as correlates to statistics anxiety in college students. Unpublished doctoral dissertation, American U niversity

[ref19] DeVellis R. F. (1991). Scale development: Theory and applications. California: Sage Publications, 1–113.

[ref20] Duval S. Tweedie R. (2000). Trim and fll: a simple funnel-plot-based method of testing and adjusting for publication bias in meta-analysis. Biometrics 56, 455–463. doi: 10.1111/j.0006-341X.2000.00455.x 10877304

[ref21] Egger M. Smith G. D. Schneider M. Minder C. (1997). Bias in meta-analysis detected by a simple, graphical test. BMJ 315, 629–634. doi: 10.1136/bmj.315.7109.629, 9310563 PMC2127453

[ref22] Erkuş A. (2011). Davranış bilimleri için bilimsel araştırma süreci. Ankara: Seçkin.

[ref23] Faber G. Drexler H. Stappert A. Eichhorn J. (2018). Education science students’ statistics anxiety: developing and analyzing a scale for measuring their worry, avoidance, and emotionality cognitions. Int. J. Educ. Psychol. 7, 248–285. doi: 10.17583/ijep.2018.2872

[ref24] Feinberg L. B. Halperin S. (1978). Affective and cognitive correlates of course performance in introductory statistics. J. Exp. Educ. 46, 11–18. doi: 10.1080/00220973.1978.11011637

[ref26] Fisch R. (1971). Course evaluation, test anxiety, and final test results in a statistics course. Z. Entwicklungspsychol. Paedagog. Psychol. 3, 212–228.

[ref27] Fitzgerald S. M. (1996). The relationship between anxiety and statistics achievement: a meta-analysis: The University of Toledo.

[ref28] Graham J. M. Christiansen K. (2009). The reliability of romantic love: a reliability generalization meta-analysis. Pers. Relat. 16, 49–66. doi: 10.1111/j.1475-6811.2009.01209.x

[ref29] Graham J. M. Diebels K. J. Barnow Z. B. (2011). The reliability of relationship satisfaction: a reliability generalization meta-analysis. J. Fam. Psychol. 25, 39–48. doi: 10.1037/a0022441, 21355645

[ref30] Hakstian A. R. Whalen T. E. Masson M. E. (1976). AK-sample procedure for comparatively assessing multivariate association. Psychol. Bull. 83, 922–927. doi: 10.1037/0033-2909.83.5.922

[ref31] Hedges L. V. (1982). Fitting categorical models to effect sizes from a series of experiments. J. Educ. Stat. 7, 119–137. doi: 10.3102/10769986007002119

[ref32] Hedges L. V. Olkin I. (2014). Statistical methods for meta-analysis. Orlando: Academic press.

[ref33] Jones A. Knibb G. Christiansen P. (2022). Statistics anxiety, predictions of exam performance and actual exam performance in UK psychology students. PsyArXiv. doi: 10.31234/osf.io/edc2n PMC10446223 37611055

[ref34] Kıyıcı G. Kahraman N. (2022). A meta-analytic reliability generalization study of the computational thinking scale. Sci. Insights Educ. Front. 13, 1859–1874. doi: 10.15354/sief.22.ma011

[ref35] Leech N. L. Onwuegbuzie A. J. O'Conner R. (2011). Assessing internal consistency in counseling research. Counsel. Outcome Res. Eval. 2, 115–125. doi: 10.1177/2150137811414873

[ref36] Light R. J. Pillemer D. B. (1984). Summing up: The science of reviewing research. Massachusetts: Harvard University Press.

[ref37] López-Pina J. A. Sánchez-Meca J. López-López J. A. Marín-Martínez F. Núñez-Núñez R. M. Rosa-Alcázar A. I. . (2015). The Yale–Brown obsessive compulsive scale: a reliability generalization metaanalysis. Assessment 22, 619–628. doi: 10.1177/1073191114551954, 25268017

[ref38] Moher D. Liberati A. Tetzlaff J. Altman D. G. Prisma Group (2009). Preferred reporting items for systematic reviews and meta-analyses: the PRISMA statement. PLoS Med. 151:e1000097. doi: 10.7326/0003-4819-151-4-200908180-00135, 19621072 PMC2707599

[ref40] O’Rourke N. Hatcher L. Stepanski E. J. (2005). A step-by-step approach to using SAS for univariate & multivariate statistics. Cary, NC, United States: SAS Institute.

[ref41] Ock J. McAbee S. T. Ercan S. Shaw A. Oswald F. L. (2021). Reliability generalization analysis of the core self-evaluations scale. Pract. Assess. Res. Eval. 26:6. doi: 10.7275/zsc7-jw58

[ref42] Orwin R. G. (1983). A fail-safe N for effect size in meta-analysis. J. Educ. Stat. 8, 157–159. doi: 10.2307/1164923

[ref43] Özdemir V. Yıldırım Y. Tan Ş. (2020). A meta-analytic reliability generalization study of the Oxford happiness scale in Turkish sample. J. Meas. Eval. Educ. Psychol. 11, 374–404. doi: 10.21031/epod.766266

[ref44] Prensky M. (2001). Digital natives, digital immigrants part II: do they really think differently? On Horizon 9, 1–6. doi: 10.1108/10748120110424843

[ref45] Rosenthal R. (1979). The file drawer problem and tolerance for null results. Psychol. Bull. 86, 638–641. doi: 10.1037/0033-2909.86.3.638

[ref1002] Sanchez‐Meca J. López‐López J. A. López‐Pina J. A. (2013). Some recommended statistical analytic practices when reliability generalization studies are conducted. British Journal of Mathematical and Statistical Psychology 66, 402–425. doi: 10.1111/j.2044-8317.2012.02057.x, 23046285

[ref46] Sánchez-Meca J. Marín-Martínez F. López-López J. A. Núñez-Núñez R. M. Rubio-Aparicio M. López-García J. J. . (2021). Improving the reporting quality of reliability generalization meta-analyses: the REGEMA checklist. Res. Synth. Methods 12, 516–536. doi: 10.1002/jrsm.1487, 33742752

[ref47] Schacht S. Stewart B. J. (1990). What's funny about statistics? A technique for reducing student anxiety. Teach. Sociol. 18, 52–56. doi: 10.2307/1318231

[ref48] Sedgwick P. (2015). Meta-analyses: what is heterogeneity? BMJ 350, 14–35. doi: 10.1136/bmj.h1435, 25778910

[ref49] Sen S. (2022). A reliability generalization meta-analysis of Runco ideational behavior scale. Creat. Res. J. 34, 178–194. doi: 10.1080/10400419.2021.1960719

[ref50] Şen S. Yıldırım İ. (2023). Meta-analysis applications with CMA. Ankara: Anı Publishing.

[ref51] Shields A. L. Caruso J. C. (2003). Reliability generalization of the alcohol use disorders identification test. Educ. Psychol. Meas. 63, 404–413. doi: 10.1177/0013164403063003004

[ref1005] Shields A. L. Caruso J. C. (2004). A reliability induction and reliability generalization study of the CAGE questionnaire. Educational and Psychological Measurement, 64, 254–270.

[ref1004] Spielberger C. D. Gonzalez-Reigosa F. Martinez-Urrutia A. Natalicio L. F. Natalicio D. S. (1970). The state-trait anxiety inventory. Revista Interamericana de Psicologia/Interamerican journal of psychology, 5.

[ref1003] Tavakol M. Dennick R. (2011). Making sense of Cronbach’s alpha. International journal of medical education, 2:53–55. doi: 10.5116/ijme.4dfb.8dfd 28029643 PMC4205511

[ref52] Taylor R. 2012 Review of the motivated strategies for learning questionnaire (MSLQ) using reliability generalization techniques to assess scale reliability. Doctoral dissertation

[ref53] Vacha-Haase T. (1998). Reliability generalization: exploring variance in measurement error affecting score reliability across studies. Educ. Psychol. Meas. 58, 6–20. doi: 10.1177/0013164498058001002

[ref54] Vahedi S. Farrokhi F. Bevrani H. (2011). A confirmatory factor analysis of the structure of statistics anxiety measure: an examination of four alternative models. Iran. J. Psychiatry 6:92, 22952530 PMC3395949

[ref55] Wallace K. A. Wheeler A. J. (2002). Reliability generalization of the life satisfaction index. Educ. Psychol. Meas. 62, 674–684. doi: 10.1177/0013164402062004009

[ref56] Yaşar M. (2014). İstatistiğe yönelik tutum ölçeği: Geçerlilik ve güvenirlik çalışması. Pamukkale Üniversitesi Eğitim Fakültesi Dergisi 36, 59–75. doi: 10.9779/PUJE640

[ref7001] Yıldırım A. B. (2021). The effect of exercise on the total number of BrdU+ cell counts in rats’ hippocampal dentate gyrus: A meta-analysis study. Brain Research, 1766, 147512. 33961895 10.1016/j.brainres.2021.147512

[ref57] Zeidner M. (1991). Statistics and mathematics anxiety in social science students: some interesting parallels. Br. J. Educ. Psychol. 61, 319–328. doi: 10.1111/j.2044-8279.1991.tb00989.x, 1786211

