# Supplementary material for: A reliability generalization meta-analysis of self-report measures of statistics anxiety
Source: Front Psychol. 2026 Jan 23;16:1675957. doi: 10.3389/fpsyg.2025.1675957 (PMC12876260; doi:10.3389/fpsyg.2025.1675957)
Supplement: Supplementary file 1 [file Supplementary_file_1.docx]

ANNEX 1.

Table S1. 81 çalışmada raporlanan 84 güvenirlik katsayısı

| ID | Author(s) | Scale | Publication Year | n of items | *n* | α | Continent | Education level | male/ female | Publication type | Mean age |
| --- | --- | --- | --- | --- | --- | --- | --- | --- | --- | --- | --- |
| 1 | Maat,2022 | STARS | 2022 | 51 | 199 | 0.960 | Asia | undergraduate |  | article |  |
| 2 | Vigil-colet,2008 | SAS | 2008 | 24 | 159 | 0.910 | Europa | undergraduate | 6.95 | article | 21.60 |
| 3 | Clark,2019 | SAS | 2018 | 14 | 323 | 0.900 | America | undergraduate | 2.99 | article | 20.64 |
| 4 | Hsiao, 2010 | STARS | 2010 | 51 | 77 | 0.940 | Asia | graduate |  | article | 27.60 |
| 5 | Seva,2022 | SAS | 2022 | 20 | 531 | 0.800 | Europa | undergraduate | 4.63 | article | 20.40 |
| 6 | Alizamar,2019 | STARS | 2018 | 51 | 368 | 0.980 | Asia | undergraduate | 3.84 | proceeding |  |
| 7 | Chiesi,2011 | SAS | 2011 | 24 | 512 | 0.900 | Europa | undergraduate | 4.28 | article | 22.30 |
| 8 | DeVaney,2016 | STARS | 2016 | 51 | 517 | 0.800 | America | undergraduate | 6.92 | article | 33.10 |
| 9 | Afdal,2019 | STARS | 2018 | 51 | 368 | 0.980 | Europa | undergraduate |  | proceeding |  |
| 10 | Steinberger,2020 | STARS | 2020 | 35 | 163 | 0.940 | Asia | undergraduate |  | article | 21.09 |
| 11 | Mji,2004 | STARS | 2017 | 51 | 196 | 0.920 | Africa | undergraduate | 2.44 | article | 21.16 |
| 12 | O'Bryant,2021 | SAS | 2021 | 24 | 323 | 0.930 | Europa | undergraduate | 1.63 | article | 20.50 |
| 13 | Ferrando,2018 | SAS | 2018 | 24 | 384 | 0.970 | Europa | undergraduate | 5.84 | article |  |
| 14 | Baloglu,2002 | STARS | 2002 | 51 | 221 | 0.960 | America | mixed | 2.88 | article | 28.00 |
| 15 | Baloglu,2003 | STARS | 2003 | 51 | 246 | 0.960 | America | undergraduate | 2.90 | article | 27.15 |
| 16 | Durak,2021 | SAS | 2021 | 24 | 439 | 0.930 | Europa | undergraduate | 1.44 | article | 21.18 |
| 17 | Papousek, 2012 | STARS | 2012 | 51 | 400 | 0.960 | Europa | undergraduate | 3.82 | article | 21.20 |
| 18 | Güler,2019 | WAESTA | 2019 | 17 | 350 | 0.960 | Europa | graduate | 1.64 | article | 30.06 |
| 19 | Liu,2011 | STARS | 2011 | 51 | 201 | 0.940 | Asia | undergraduate |  | article |  |
| 20 | Pretorius,1992 | SAS-10 | 1992 | 10 | 337 | 0.900 | Africa | undergraduate | 1.39 | article | 22.08 |
| 21 | Hsiao,2011 | STARS | 2011 | 51 | 77 | 0.940 | Asia | graduate | 4.50 | article | 27.56 |
| 22 | Lavasani,2014 | STARS | 2014 | 51 | 345 | 0.900 | Asia | undergraduate |  | proceeding |  |
| 23 | Wu,2022 | STARS | 2022 | 51 | 301 | 0.960 | Asia | undergraduate | 1.25 | article | 19.54 |
| 24 | MacArthur,2023 | STARS | 2023 | 51 | 111 | 0.940 | America | undergraduate | 2.47 | article |  |
| 25 | Sandoz,2017 | SAS | 2017 | 24 | 139 | 0.920 | America | undergraduate |  | article |  |
| 26 | Faber,2018 | WAESTA | 2018 | 17 | 113 | 0.940 | Europa | graduate | 4.95 | article |  |
| 27 | Gibeau,2023 | SAS | 2023 | 21 | 773 | 0.950 | Europa & Africa | undergraduate | 5.84 | article | 22.10 |
| 28 | McIntee,2022 | SAS | 2022 | 21 | 238 | 0.930 | America | undergraduate | 4.53 | article | 21.85 |
| 29 | Van Dijck,2022 | SAS | 2022 | 24 | 438 | 0.910 | Europa | undergraduate | 5.84 | article | 18.23 |
| 30 | Zhang,2021 | STARS | 2021 | 51 | 90 | 0.790 | America | undergraduate |  | article | 23.13 |
| 31 | Faber,2019 | WAESTA | 2019 | 17 | 87 | 0.940 | Europa | graduate |  | article |  |
| 32 | Macher,2013 | STARS | 2013 | 23 | 284 | 0.930 | Europa | undergraduate | 3.81 | article | 21.40 |
| 33 | Beurze,2013 | STARS | 2013 | 51 | 276 | 0.940 | Europa | undergraduate | 2.03 | article |  |
| 34 | Hamid,2016 | STARS | 2016 | 51 | 139 | 0.900 | Asia | undergraduate |  | proceeding |  |
| 35 | Obryant,2017 | SAS | 2017 | 24 | 323 | 0.930 | America | undergraduate | 2.85 | dissetation | 20.50 |
| 36 | Maat,2016 | STARS | 2016 | 51 | 173 | 0.920 | Asia | undergraduate | 3.55 | article |  |
| 37 | Paul,2018 | SAS | 2018 | 24 | 238 | 0.870 | Asia | undergraduate | 0.80 | article | 21.53 |
| 38 | MaySick,1985 | STARS | 1985 | 51 | 350 | 0.970 | America | undergraduate | 1.00 | thesis |  |
| 39 | Altun,2021 | WAESTA | 2022 | 17 | 101 | 0.960 | Europa | graduate | 2.96 | article |  |
| 40 | Altun,2021(2) | SAS | 2022 | 17 | 101 | 0.900 | Europa | graduate | 2.96 | article |  |
| 41 | Grajzel,2019 | STARS | 2019 | 51 | 275 | 0.890 | America | mixed | 2.67 | thesis |  |
| 42 | Grajzel,2019(2) | SAS | 2019 | 24 | 275 | 0.940 | America | mixed | 2.67 | thesis |  |
| 43 | Vink,2017 | STARS | 2017 | 23 | 50 | 0.930 | Europa | undergraduate | 6.14 | thesis |  |
| 44 | Idika,2020 | SAQ | 2020 | 35 | 943 | 0.780 | Africa | undergraduate | 1.01 | article | 29.87 |
| 45 | Casinillo,2022 | SAS-10 | 2022 | 10 | 120 | 0.750 | Asia | undergraduate |  | article | 20.10 |
| 46 | Igbokwe,2017 | STARS | 2017 | 51 | 260 | 0.960 | Africa | undergraduate | 1.36 | article | 20.97 |
| 47 | Igbokwe,2017(2) | SAS | 2017 | 24 | 260 | 0.910 | Africa | undergraduate | 1.36 | article | 20.97 |
| 48 | İbrahim Ali,2022 | STARS | 2022 | 17 | 200 | 0.800 | Asia | undergraduate |  | article | 28.71 |
| 49 | Earp,2007 | SAM | 2007 | 23 | 347 | 0.930 | America | mixed | 1.50 | thesis | 24.35 |
| 50 | Pan,2004 | SAS-10 | 2004 | 10 | 21 | 0.980 | America | graduate | 9.50 | article | 37.00 |
| 51 | Primi,2018 | SAS | 2018 | 24 | 138 | 0.940 | Europa | undergraduate | 3.93 | proceeding | 20.81 |
| 52 | Watson,2003 | STARS | 2003 | 36 | 69 | 0.950 | America | graduate |  | proceeding |  |
| 53 | Mji,2009 | STARS | 2009 | 51 | 226 | 0.880 | Africa | undergraduate | 1.97 | proceeding | 20.10 |
| 54 | Perepiczka,2011 | STARS | 2011 | 51 | 166 | 0.880 | Europa | graduate | 4.53 | article | 34.40 |
| 55 | Watson,2002 | STARS | 2002 | 51 | 69 | 0.940 | America | graduate |  | proceeding | 39.00 |
| 56 | Mandap,2016 | STARS | 2016 | 51 | 180 | 0.950 | Asia | undergraduate | 1.37 | article |  |
| 57 | Ogbogo,2018 | STARS | 2018 | 51 | 150 | 0.920 | Africa | undergraduate | 0.70 | article |  |
| 58 | Onwuegbuzie,1997 | STARS | 1997 | 51 | 81 | 0.960 | America | graduate | 6.36 | article | 31.30 |
| 59 | Lin,2017 | STARS | 2017 | 51 | 113 | 0.810 | America | mixed |  | article |  |
| 60 | Yulianto,2022 | STARS | 2022 | 19 | 73 | 0.840 | Asia | undergraduate | 3.06 | article | 19.35 |
| 61 | Hoegler,2018 | STARS | 2018 | 51 | 63 | 0.950 | America | undergraduate | 4.73 | article | 21.62 |
| 62 | Mokhele,2018 | STARS | 2018 | 51 | 103 | 0.740 | Africa | undergraduate | 1.06 | thesis |  |
| 63 | Rosli,2017 | STARS | 2017 | 51 | 199 | 0.960 | Asia | undergraduate | 3.42 | article |  |
| 64 | McGrath,2015 | STARS | 2015 | 18 | 28 | 0.870 | America | graduate |  | article | 27.15 |
| 65 | Petkovska,2020 | STARS | 2020 | 51 | 117 | 0.910 | Europa | undergraduate | 1.39 | proceeding | 20.63 |
| 66 | Bell,2022 | STARS | 2022 | 51 | 219 | 0.970 | America | graduate | 0.81 | thesis |  |
| 67 | Onwuegbuzie,1993 | STARS | 1993 | 51 | 26 | 0.960 | America | graduate | 4.20 | thesis | 40.73 |
| 68 | Baloglu,2004 | STARS | 2004 | 51 | 105 | 0.880 | America | mixed | 4.25 | article | 28.00 |
| 69 | Kangni,2021 | STARS | 2021 | 51 | 66 | 0.910 | Europa | undergraduate | 1.13 | thesis | 23.85 |
| 70 | Heretick,2021 | STARS | 2021 | 51 | 107 | 0.980 | America | undergraduate |  | article |  |
| 71 | Liu,2021 | SAS | 2021 | 27 | 709 | 0.950 | America | mixed | 1.36 | article |  |
| 72 | Lalande,2019 | SAS | 2019 | 24 | 247 | 0.910 | America | undergraduate | 4.88 | article | 21.82 |
| 73 | Kafadar,2022 | SAS | 2022 | 24 | 58 | 0.900 | Europa | graduate | 1.07 | article |  |
| 74 | Fabbricatore,2022 | SAS | 2022 | 24 | 201 | 0.790 | Europa | undergraduate | 5.09 | article | 19.70 |
| 75 | Neumann,2009 | STARS | 2009 | 51 | 52 | 0.940 | Australia | undergraduate |  | article | 22.63 |
| 76 | Onwuegbuzie,2000 | STARS | 2000 | 51 | 146 | 0.890 | America | graduate | 10.23 | article | 31.50 |
| 77 | Dunn,2014 | STARS | 2014 | 51 | 101 | 0.900 | America | graduate | 2.88 | article | 33.00 |
| 78 | Larwin,2012 | STARS | 2012 | 23 | 238 | 0.920 | America | graduate |  | article |  |
| 79 | Bui,2011 | STARS | 2011 | 51 | 104 | 0.750 | America | undergraduate | 3.30 | article | 23.42 |
| 80 | Teman,2013 | STARS | 2013 | 51 | 423 | 0.960 | America | mixed | 2.25 | article | 26.60 |
| 81 | Yeşildağ,2022 | WAESTA | 2022 | 17 | 44 | 0.950 | Europa | graduate | 0.83 | article |  |
| 82 | Yunis,2011 | STARS | 2016 | 47 | 164 | 0.930 | Africa | undergraduate | 12.67 | article | 19.03 |
| 83 | Bathurst,2015 | STARS | 2015 | 51 | 54 | 0.940 | America | undergraduate |  | thesis | 21.37 |
| 84 | Sockol,2021 | SAS-10 | 2021 | 10 | 91 | 0.930 | America | undergraduate | 5.00 | article |  |
